# Virtual Maternity Care During Pregnancy: A Metasynthesis of the Qualitative Literature on Women’s Experiences

**DOI:** 10.3390/ijerph23050607

**Published:** 2026-05-04

**Authors:** Jennifer Fenwick, Olga Aleshin, Jennifer Green, Vanessa Scarf, Heike Roth, Kathleen Baird, Helen Barrett, Deborah Fox

**Affiliations:** 1 Collective of Midwifery and Child and Family Health, University of Technology Sydney, Ultimo 2007, Australia; jennifer.fenwick@uts.edu.au (J.F.); olga.aleshin@student.uts.edu.au (O.A.);; 2 The Royal Hospital for Women, Randwick 2031, Australia

**Keywords:** virtual maternity care, antenatal care, telehealth, remote monitoring, women’s experiences, qualitative metasynthesis, health equity, continuity of care, digital health

## Abstract

**Highlights:**

**Public health relevance—How does this work relate to a public health issue?**

- Virtual maternity care is increasingly used worldwide to improve access, efficiency, and continuity of antenatal services, making women’s experiences a critical public health concern.
- Understanding how virtual care shapes perceived safety, engagement, and equity is essential as health systems scale digital models beyond pandemic contexts.

**Public health significance—Why is this work of significance to public health?**

- This metasynthesis provides a conceptually rich synthesis of women’s experiences, identifying relational continuity, technological reliability, and hybrid care as central to safe and acceptable virtual maternity services.
- The findings highlight how poorly designed virtual models risk exacerbating existing health inequities, particularly for women experiencing digital exclusion or psychosocial vulnerability.

**Public health implications—What are the key implications or messages for practitioners, policy makers and/or researchers in public health?**

- Public health strategies should prioritise hybrid models of maternity care that integrate virtual services with relationship-based, in-person support to promote safety, equity, and engagement.
- Policy and research must explicitly address digital inclusion, workforce capability, and relational care to ensure virtual maternity care strengthens rather than fragments public health systems.

**Abstract:**

The adoption of Virtual Maternity Care (VMC) in antenatal settings is increasing, propelled by technological advancements that facilitate remote communication and telemonitoring. A spectrum of care models exists globally, ranging from fully virtual to hybrid approaches. This review seeks to synthesise the qualitative evidence on women’s experiences of antenatal VMC in high-income countries, developing a conceptually rich understanding of factors that facilitate or hinder engagement and perceived safety. In June 2025, four databases were searched for peer-reviewed literature published in English between January 2010 and June 2025. After screening for quality and eligibility, 21 articles were included. Four core themes were identified: Virtual Care Worked Well, Seeking Good Connections, Empowerment and Safety Through Virtual Care Monitoring, and Feeling Disconnected and Unsafe. Women described feeling empowered through active participation and shared responsibility in their care (Empowerment and Safety Through Virtual Care Monitoring), particularly when relational care and continuity were present (Seeking Good Connections). Flexibility, convenience, and access to daily telehealth and reliable technologies were highly valued (Virtual Care Worked Well). Hybrid models were generally preferred; in contrast, exclusively remote models sometimes inhibited quality care and heightened feelings of insecurity (Feeling Disconnected and Unsafe), particularly for women with previous pregnancy loss, experiences of intimate partner violence, mental health concerns, or those facing language barriers, digital poverty, financial hardship, or low health literacy. In conclusion, women’s perspectives underscore priorities for designing and scaling high-quality, accessible virtual midwifery care: relational continuity, technological reliability, flexible delivery, and hybrid models integrating virtual and in-person care.

## 1. Introduction

The use of Virtual Maternity Care (VMC) in antenatal settings is expanding rapidly worldwide, driven by innovations in technology that support remote communication and telemonitoring. The implementation of VMC in antenatal settings has evolved into a spectrum of models that reflect varying degrees of virtual engagement. At one end of this spectrum are fully virtual models, where antenatal care is delivered exclusively through telehealth platforms [1]. These models rely on phone or video consultations and may include remote monitoring technologies, allowing some or all routine antenatal visits to be conducted virtually. Such approaches are particularly beneficial for women who face barriers to accessing in-person care, whether due to geography, mobility, or personal circumstances [2]. In contrast, hybrid models blend virtual and face-to-face care [3,4]. These models typically involve scheduled in-person visits, either at home, in clinics, or hospital settings, complemented by virtual consultations. The hybrid approach aims to maintain clinical safety while offering flexibility and convenience, often reserving in-person care for physical assessments or when complex clinical needs arise [5].

Within both fully virtual and hybrid models, remote monitoring plays a central role. Two distinct forms of monitoring are commonly used: remote self-monitoring and telemonitoring. Remote self-monitoring requires women to use home-based equipment to track specific clinical parameters such as blood pressure, heart rate, temperature, and glucose levels. These measurements are then shared with care providers, either during live consultations or through asynchronous communication channels such as secure messaging or smart application uploads [6]. Telemonitoring involves the use of integrated digital systems that automatically transmit clinical data to healthcare providers. This method enables continuous or scheduled monitoring of maternal and fetal health indicators, including blood pressure, glucose levels, maternal heart rate, fetal heart rate patterns, and uterine activity. The seamless transmission of data supports timely clinical decision-making and enhances the responsiveness of care [7].

The implementation of antenatal VMC varies globally, shaped by differing health system structures, policies, and technological capacities. During the COVID-19 pandemic, many virtual healthcare models were rapidly introduced in response to social distancing requirements and heightened infection control measures [8]. There is value in examining the impact of these models on women’s experiences, particularly for health organisations seeking to develop and implement sustainable antenatal VMC. While existing reviews have examined the uptake, effectiveness, and clinical outcomes of digital or virtual antenatal care, these have predominantly focused on feasibility, implementation, and service-level outcomes rather than synthesising women’s lived experiences across models of care [9]. At the time of the review, no published metasyntheses could be identified that consolidated qualitative literature on women’s experiences of antenatal VMC.

The aim of this metasynthesis was to interpret and synthesise qualitative evidence on women’s experiences of virtual maternity care in high-income countries to develop a conceptually rich understanding of the factors that facilitate or hinder engagement and perceived safety.

In this manuscript, “virtual maternity care” (VMC) is used as an umbrella term to describe digitally mediated models of antenatal care, encompassing telehealth consultations, remote self-monitoring, and telemonitoring systems. Where possible, more specific terms are used to reflect the modality being discussed.

## 2. Methodology

This study was theoretically grounded in an interpretivist perspective, recognising that meaning is constructed through context and interpretation, rather than being reducible to isolated findings. A qualitative synthesis of the literature was conducted using the meta-ethnography approach developed by Noblit and Hare [10]. This method has been widely applied to synthesise qualitative research in maternity care [11,12] and across other health disciplines [13], supporting the development of high-quality evidence to inform healthcare interventions and models of care. To support conceptual transferability within comparable care contexts, the synthesis focused on high-income health systems with similar digital infrastructure and maternity service organisation, while recognising this as a limitation for global generalisation.

### 2.1. Reporting Standards

This systematic review and qualitative metasynthesis was conducted and reported in accordance with the Preferred Reporting Items for Systematic Reviews and Meta-Analyses (PRISMA) 2020 guidelines [14]. The PRISMA 2020 checklist is provided in Supplementary File S1. The review protocol was not prospectively registered, as protocol registration is not mandatory for qualitative metasyntheses and the review adopted an iterative, inductive analytic approach; to support transparency, all methodological decisions are reported in full.

### 2.2. Search Strategy

The literature search was conducted in June 2025 by two authors, OA and JG, with support from an expert university librarian. Peer-reviewed literature published in English between 1 January 2010, and 3 June 2025 was sought. The aim was to identify studies that provided qualitative data from women who had experienced virtual maternity care in high income countries. Medline, EMBASE, CINAHL and Scopus databases were searched, using key terms grouped under four main headings including ‘virtual’, ‘maternity care’, ‘experience’ and ‘women/pregnant people’. The full electronic search strategies, including all search terms and combinations used for each database, are provided in Supplementary File S2.

### 2.3. Inclusion and Exclusion Criteria

Studies published in English that reported qualitative findings on women’s experiences of VMC during pregnancy were included. Studies conducted in low- and middle-income countries (LMICs) were excluded, not due to a lack of relevance or importance, but to support conceptual coherence and analytic depth within the meta-ethnographic synthesis. Virtual maternity care is shaped by health-system infrastructure, digital connectivity, workforce configuration, and models of antenatal service delivery, which differ markedly across economic and policy contexts. Meta-ethnography requires comparison across studies with sufficient contextual similarity to allow meaningful translation of concepts; combining LMIC and high-income country studies risked masking structurally driven differences and producing over-generalised interpretations. Focusing on high-income settings therefore enabled more rigorous conceptual synthesis, while recognising that women’s experiences of virtual maternity care in LMIC contexts warrant dedicated, context-specific synthesis in future research. Studies focusing on the perspectives of healthcare professionals or women’s family members were also excluded, as the aim of this synthesis was to centre the voices and experiences of women themselves.

**Figure 1:**
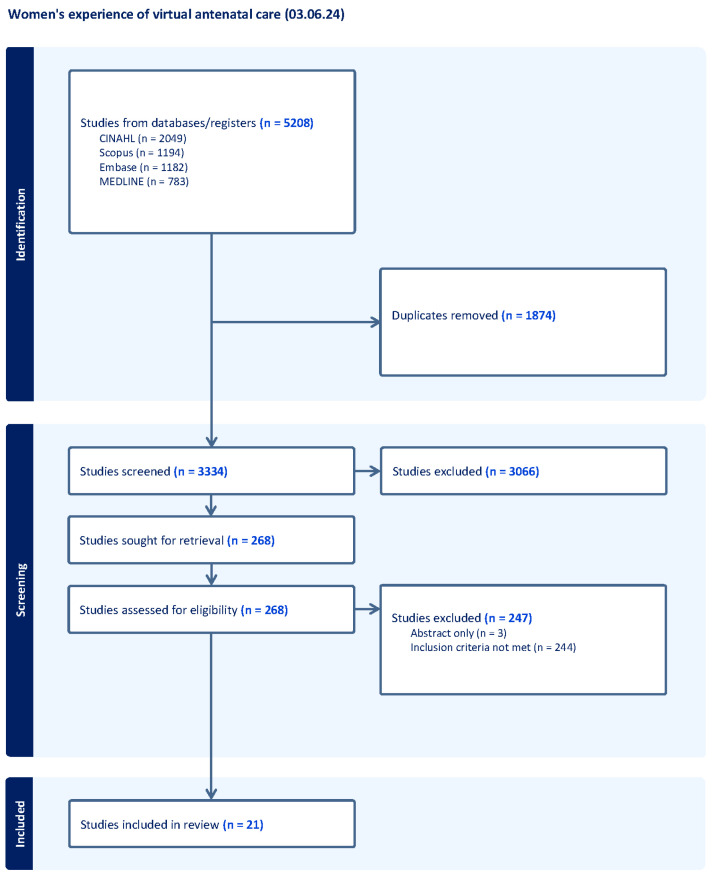
PRISMA 2020 flow diagram illustrating the study selection process. The diagram was generated using Covidence systematic review software.

The initial database search revealed 5208 studies. Covidence systematic review software (2025) [15] was utilised to store the search records and manage the collaborative screening process across the co-authorship team. After duplicates were removed, 3334 studies were screened for eligibility based on the criteria listed above, resulting in 268 studies for abstract and full-text review. This process was conducted by seven authors with 247 articles excluded. Study quality was independently assessed by two reviewers using the Critical Appraisal Skills Programme (CASP) qualitative checklist, with discrepancies resolved through discussion. All 21 studies were retained. The Preferred Reporting Items for Systematic Reviews and Meta-Analyses (PRISMA) guidelines informed the process of reporting the search strategy [14]. Figure 1 outlines the search strategy using the PRISMA diagram.

### 2.4. Data Characteristics

Table 1 summarises the characteristics of the 21 included studies, detailing their method, design, setting, participant numbers, and application of virtual maternity care. This table supported the translation of concepts across studies and provided context for the synthesis of women’s experiences and concepts raised in the authors analyse.

The total of 21 included studies represented the perspectives of 369 women who had experienced VMC during pregnancy. Fourteen were qualitative studies, and seven were mixed methods studies from which only the qualitative findings were extracted. Seven studies were conducted in the United States (US) [16,17,18,19,20,21,22], six in the United Kingdom [5,23,24,25,26,27], four in Scandinavia [7,28,29,30], two in The Netherlands [31,32] and two in Australia [4,33]. One study collected data across both Northern Ireland and the Republic of Ireland [23].

Each study contained its own terminology for discussing the various applications of VMC, many terms are used interchangeably. Table 2 Glossary provides a list of definitions that have been adopted for this project.

The studies included in this review applied VMC in various ways within antenatal outpatient settings (clinical visits via telehealth throughout pregnancy) or antenatal inpatient/Hospital at Home settings (monitoring of pregnancy complications as an alternative to admission to hospital). Table 3 provides details of the different settings described.

**Table 1:** Characteristics of included studies.

Author, Date, Country	Aim of the Study	Study Design and Methodology Participants	VMC Application/Mode	Themes/Categories of Qual Data as Determined by the Authors
Altman et al., 2023, USA [16]	To describe pregnant women and birthing people’s experiences of virtual care during pregnancy, birth, and postpartum among a diverse group in Washington State during COVID.	Inductive qualitative design. In-depth interviews, critical thematic analysis, critical discourse analysis, situational analysis. Pregnant and birthing people (n = 15).	Virtual care during pregnancy.	*Loss of connection and relationships with* *providers.* *Need for hands-on interactions for reassurance.* *Virtual care is good for some things but not all—desire for immediate, accessible care when appropriate.*
Andreasen et al., 2024, Denmark [28]	To understand the barriers and facilitators experienced by Danish pregnant women that influenced their participation in digital IPV screening and the digital supportive ‘STOP’ intervention.	Qualitative thematic analysis. Semi-structured interviews Pregnant women <25 weeks gestation, screened positive for IPV (n = 20).	Online screening for IPV and delivery of digital intervention (the STOP Study).	*Facilitators and barriers related to digital screening.* *Facilitators and barriers for participating in digital supportive IPV intervention.* *Areas for improvement.*
Bachiller et al., 2024, USA [17]	To understand attitudes towards telemedicine and to further elucidate benefits, disadvantages, and visit preferences in a largely minority, urban safety-net setting	Modified grounded theory and content analysis. In-depth interviews. Ethnically diverse population in low SES setting (n = 42).	VMC replacing some Antenatal outpatient visits with hybrid AV, phone and f2f.	*Perceived benefits of telehealth.* *Perceived disadvantages of telehealth.* *Telemedicine* vs. *in-person visit preferences.* *Video* vs. *audio telemedicine visits.* *Telemedicine preferences post-pandemic.*
Bendix et al., 2024, Denmark [7]	To examine how women with pregnancy complications experienced performing home-based telemonitoring	Qualitative. Methodology based on Malterud’s systematic text condensation and Giorgi’s psychological phenomenological theory (n = 15 women).	VMC replacing inpatient admission (Hospital at Home) with telemonitoring, telehealth by phone. Data collected during COVID-19, but model was established.	*Empowering yet challenging responsibility.* *Extended patient-clinician partnership.* *Tele-comfort yet ambivalence.* *Accompanying remote issues.*
Collins et al., 2024, Australia [33]	To examine childbearing women and midwives’ experiences of using telehealth during the COVID-19 pandemic.	Mixed methods study. Qualitative interviews and open-ended survey responses collected from March 2020-December 2020. Content analysis approach. Pregnant or postpartum women who had given birth during COVID-19 were interviewed (n = 20). Open text responses from survey (n = 812).	VMC replacing antenatal outpatient visits with hybrid AV, synchronous maternity care by telephone or video.	*Women were let down by the system.* *Telehealth was beneficial for some women but not all.* *Inconsistency in telehealth.* *Limitations to technology.*
Farrell et al., 2022, USA [18]	To examine prenatal care needs, preferences, and experiences during the COVID-19 pandemic.	Inductive qualitative approach consistent with grounded theory method of Corbin and Strauss. In depth telephone interviews (n = 40).	Telehealth (not defined).	*Perceptions of the benefits of telehealth during the pandemic.* *Reassurance that comes from in-person clinical visits with an obstetric provider.* *Added concerns about the responsibility of determining the well-being of the pregnancy at home.* *The impact of telehealth on patient experience with pregnancy and prenatal care.*
Given et al., 2015, Northern Ireland & Republic of Ireland [23]	To determine feasibility and acceptability of using telemedicine with women with GDM to replace alternate (one in every two) diabetes review appointments with telemedicine.	RCT of telemedicine/usual care for women with GDM. Qualitative interviews with intervention group (n = 24).	Telemedicine “hub” installed in the woman’s home. Weekly virtual consults using BGL, BP and weight data collected sent via telemedicine hub.	*Potential benefits with telemedicine.* *Using the telemedicine equipment.* *Closer monitoring.* *What would be lost.*
Hinton et al., 2023, England [24]	To explore the views and experiences of women, healthcare providers and system leaders about remote antenatal care, using the lens of candidacy as a framework for analysis.	Qualitative study using theoretical perspective of candidacy framework for understanding influences on access to health care. Qualitative semi structured Interviews using constant comparative method (n = 45 women).	VMC replacing antenatal outpatient visits during COVID. AV, phone. Hybrid face to face and virtual.	*Women’s identification of candidacy for themselves and their baby.* *Navigation.* *Permeability of services.* *Appearing at services.* *Adjudications.* *Operating conditions and the local production of candidacy.*
Hinton et al., 2024, England [5]	To characterise what quality would look like for remote antenatal care from the perspectives of those who use, provide and organise it.	Qualitative check methodology with semi structured interviews. Constant comparative method used. Survey with free-text responses and semi-structured interviews (n = 45 women).	VMC replacing antenatal outpatient visits during COVID. AV, phone. Hybrid face to face and virtual.	*Efficiency and timeliness.* *Effectiveness.* *Safety.* *Accessibility.* *Equity and inclusion.* *Person- centredness.* *Choice and continuity.*
Howard et al., 2023, USA [19]	To identify the facilitators and barriers to receiving perinatal telepsychiatry care from the perspective of patients (n = 8), clinic staff and psychiatrists.	Qualitative semi structured interviews (n = 8 women).	Specialised perinatal mental health or substance use, replacing outpatient visits. The intervention was a one-time assessment, after which the psychiatrist contacted the GP with a treatment plan. AV/phone.	*Advantages to telepsychiatry.* *Barriers to telepsychiatry care.* *The importance of communication between care staff and patients.* *The use of technology to facilitate appointment attendance.* *Suggestions for improving telepsychiatry access.*
Jepsen et al., 2024, Denmark [29]	To investigate how women with complicated pregnancies experienced telemonitoring of the fetal heart rate.	Qualitative design, reflexive thematic analysis: Braun & Clarke thematic analysis. Women with gestational diabetes or Type 1 Diabetes Mellitus (n = 11).	Telemedicine (not defined).	*Time management.* *Comparing telemonitoring with hospital visits.* *Technical challenges.* *Feelings about telemonitoring.* *The need for feedback.*
Jones et al., 2023, USA [20]	To measure patients’ satisfaction with and feasibility of using an integrated model of cellular-enabled RPM devices for BP supported by a 24/7 nurse call centre.	Mixed-methods study using a pre-post survey. design and semi-structured qualitative interviews. Women with hypertension in pregnancy (n = 20).	Women used a cellular-enabled BodyTrace BP cuff. BP was constantly monitored.	*Advantages: perceptions that their care was better; BP being remotely monitored by a health professional rather than exclusively by the patients themselves; increased participant empowerment; convenience; ease of use of the device.* *Disadvantages: issues with the device; issues with the Response Protocol*
Jones et al., 2024, USA [21]	To assess maternal and neonatal clinical outcomes as well as patient acceptability of an integrated model of cellular-enabled remote patient monitoring (RPM) devices for BP supported by a 24/7 nurse call centre.	Mixed method study: pre-post surveys, qualitative semi-structured telephone interviews (n = 20 women).	Women used a cellular-enabled BodyTrace BP cuff and weight scales. BP readings were automatically uploaded to a physician portal.	*Advantages: easy/convenient to use; perceived better care; increased monitoring of BP; call center support; participant empowerment.* *Disadvantages: issues with protocol; inaccurate readings*
Jongsma et al., 2020, The Netherlands [31]	To explore the experiences of Dutch pregnant women who used a hybrid approach (mHealth and f2f) for remote self-monitoring of BP and preeclampsia symptoms	Mixed methods design (questionnaires and interviews) Pregnant women at increased risk of HPD were interviewed (n = 11).	Safe@Home study using automated blood pressure monitor with Bluetooth connection to a smartphone app. Data was reviewed 5 days a week by digital monitoring team (midwife or nurse). Face to face appointments continued, with an adjusted schedule.	*Expectations of and satisfaction with the mHealth technology.* *Usability of the mHealth tool.* *Autonomy and responsibility of patients.* *Health care professionals’ expertise and responsibilities.*
Kissler et al., 2024 USA [22]	To describe patients’ and providers’ experiences with telehealth during the COVID-19 pandemic, to inform future utilization of telehealth towards high-quality, accessible, and equitable care to diverse communities.	Descriptive exploratory qualitative study. Two rounds of semi-structured interviews with pregnant women (n = 14).	Virtual visits were conducted synchronously with 2-way video conferencing using an electronic health record–based system in place prior to the pandemic. In-person visits were included particularly in third trimester.	*Unexpected advantages of telehealth.* *Patient empowerment.* *Providers’ fear of adverse outcomes.* *Concern for equitable care.* *Strategies to enhance the telehealth experience.* *Strategies to address access to perinatal telehealth.*
Kozica-Olenski et al., 2022, Australia [4]	To explore the experiences of telehealth in diabetes in pregnancy care and general pregnancy care during the COVID-19 pandemic, from the perspectives of pregnant women and clinicians.	Qualitative study in-depth interviews using a thematic inductive approach. Culturally and linguistically diverse pregnant women (n = 18).	Women received their maternity care via a combination of telephone, videoconferencing and face-to-face consultations.	Authors used the seven domains of the NASSS framework by Greenhalgh et al. [34] to structure the analysis: *Condition* *Technology* *Value proposition* *Adopters* *Organisations* *Wider system* *Embedding and adaptation over time*
Nääs et al., 2025, Sweden [30]	To explore women’s experiences of participating in a digital continuity of care model designed for pregnant women with fear of birth.	Qualitative design using reflexive thematic analysis (n = 15 women).	Project midwives provided counselling support and birth planning digitally via an e-health tool (video link with face-to-face interactions).	*Overarching theme: A digital continuity model of midwifery care for women with fear of birth in a rural area is attractive.* *Themes:* *A way to create positive outcomes in terms of sustainability and use of resources.* *Continuity of care—A means to create confidence and security during the entire process of childbirth.* *The importance of having access to a midwife.* *A way to meet women’s unique needs.*
Paterson et al., 2023, Scotland [25]	1. To explore the way in which the supported self-monitoring programme was implemented across contrasting sites 2. To assess the views and experiences of women and staff participating in the supported self-monitoring of BP programme	Qualitative case series study, using semi-structured telephone interviews with pregnant women (n = 20).	Rapid roll-out of supported self-monitoring of BP.	*Outcomes (clinical outcomes, service outcomes, psychosocial outcomes).* *Barriers and facilitators to implementation.*
Pilav et al., 2022, England [26]	To explore minority ethnic women’s experiences of perinatal mental health services during first wave of COVID-19 in London.	Qualitative study design, semi-structured interviews (n = 18 women).	Perinatal mental health support provided remotely, by either audio visual or telephone modalities.	*Difficulties and disruptions to access.* *Experiences of remote delivery.* *Psychosocial experiences linked to COVID-19.*
van den Heuvel et al., 2020, The Netherlands [32]	To explore the views and experiences of women about being admitted (one group) OR being telemonitored at home during pregnancy (one group)	Qualitative design, focus groups Postpartum women: 11 who were admitted during pregnancy and 11 who experienced telemonitoring (n = 22).	Wireless devices for blood pressure (Microlife watchBP) and cardiotocography (Sense4Baby, BMA- Telenatal, The Netherlands) were used for daily follow up of patients with either PPROM, FGR or preeclampsia.	*Care experience.* *Emotions regarding pregnancy.* *Privacy.* *Impact on daily life.*
Wilson et al., 2022, England [27]	To evaluate maternity units’ implementation (and women’s experience) of self-monitoring BP during COVID-19	Mixed methods design: surveys, anonymised patient data and in-depth interviews with women (n = 23).	Self-monitoring of BP.	*Experiences and effects of self-monitoring of blood pressure.* *Remote vs. face-to-face appointments.* *App and telemonitoring.*

**Table 2:** Glossary.

Term	Definition	Reference
App or application	A software which is downloaded onto a mobile device and may be used for communication or collection of health data.	[35]
Artificial Intelligence	The ability of a computer system to perform tasks commonly associated with intelligent beings, such as learning, decision-making and reasoning.	[36]
Device or ‘smart’ device	A device is the tool used to access healthcare information and data, such as a mobile phone, tablet or computer.	[35]
	A ‘smart’ device is an electronic device which uses an internet connection to send and receive data.	[37]
Digital health	Using technology to develop systems, tools and services for healthcare purposes. It is an umbrella term for all digital technologies in healthcare. This includes (but is not limited to), mobile health and applications, digital medicines and electronic health records, telehealth, wearable devices, robotics and artificial intelligence.	[38]
Digital literacy	Underpinned by basic skills in using information and communications technology, digital literacy involves the ability of individuals to safely, appropriately and confidently use digital technologies in various aspects of life.	[39]
Digital platform	The software used to connect healthcare providers and healthcare consumers for online consultations or interactions.	[35]
Electronic health or eHealth (E-Health)	A sub-component of digital health, eHealth specifically refers to the use of information and communications technology for healthcare.	[1]
Electronic Medical Record (eMR)	A person’s medical record in digital form.	[40]
Home-based care or home visiting	The provision of care from health professionals to consumers in the consumers home/residence. The care may be provided on a full-time basis, or via scheduled visits, depending on the individual’s needs.	[3]
Hospital in the Home (HiTH) or Hospital at Home (HaH/H@H)	Also referred to as ‘Hospital at Home’, Hospital in the Home is a model of care where consumers receive acute, hospital-level care in their home/residence. This reduces the length of hospital admission, or the need for admission altogether, as well as outpatient visits to hospital.	[40]
Hybrid care or blended care	Also referred to as blended care, hybrid care refers to the provision of healthcare via both in-person and virtual modalities. In this type of care, face-to-face care is combined with care either via phone or video consultations.	[35,41]
Hybrid model/service	A hybrid model or service is a model of care which implements hybrid/blended care as described above.	
Information and Communications Technology (ICT)	ICT refers to the integration of IT (see below) with other technologies such as broadcasting and telecommunications to assist with access to information and connectivity.	[42]
In-person care or face-to-face care	Also referred to as face-to-face care, in-person care refers to healthcare provided in the traditional sense, where the health provider and consumer are both present in the same physical location.	[41]
Interoperability	The ability to share information between people, organisations and systems in a manner that is easy, safe and secure.	[43]
Information Technology (IT)	Refers to the use of hardware (such as computers), software, databases and networks to process, manage and store data.	[42]
Mobile Health (mHealth)	Mobile health (mHealth) refers to the use of mobile and wireless devices to support healthcare.	[44]
Remote monitoring or telemonitoring	Also referred to as telemonitoring, remote monitoring is a method of gathering health data from a consumer at home. This is usually done using an app or device, and the information is virtually reviewed by a healthcare provider. Examples include remote fetal heart rate monitoring, remote blood pressure monitoring and remote glucose monitoring. Some of these monitoring devices do not use smart technology and are reported by consumers directly to healthcare providers.	[41]
Secure messaging	The use of secure, electronic technology (such as apps or SMS) for quick and convenient communication between healthcare providers and consumers. May also be referred to as digital messaging.	[45]
Self-measurement or Self-monitoring	Like remote monitoring, self-measurement (also referred to as self-monitoring) refers to the process where the consumer uses digital tools and/or technology to collect specific healthcare data, which is then reviewed remotely by a healthcare provider. This could also involve no device, e.g., self-monitoring of symptoms.	[46]
Telecommunication technologies	Using electronic means to transmit and receive information over long distances. Examples include phones, internet and radio.	[47]
Telehealth	Using technology such as phone or video to remotely access or provide a wide range of healthcare services (both clinical and non-clinical). See also virtual care.	[40]
Telemedicine	A subset of telehealth, telemedicine refers to the use of technology to support the remote provision of medical, diagnostic and treatment related healthcare.	[48]
Videoconferencing	The use of video to connect two or more people, allowing them to see and hear each other.	[40]
Virtual consultation or remote consultation	Also referred to as a remote consultation, a virtual consultation is where a healthcare provider and consumer meet to discuss care via videoconferencing technology.	[40]
Virtual health or virtual care	Also known as telehealth, virtual health utilises technology to assist the provision of healthcare to consumers remotely. May also be referred to as virtual care.	[40]
Virtual hospital	Represents a healthcare service that operates solely in the digital realm.	[49]
Virtual Ward—see also Hospital in the Home	A hospital-led service, where consumers who would otherwise be admitted to hospital, receive acute, hospital-level care in their home/residence, enabled by technology.	[50]
Wearables	Electronic devices worn by individuals to continuously monitor and transmit health data for clinicians to review in real time.	[51]

**Table 3:** Variations in VMC Provision.

Outpatient Settings	Routine antenatal visits are attended in a hybrid format, virtually and in-person. +/− Remote self-monitoring of maternal/fetal parameters.
	Routine antenatal visits are attended virtually. In-person visits are attended if there is identification that escalation of care is required. +/− Remote self-monitoring of maternal/fetal parameters.
	Routine antenatal visits are attended in-person, specialised visits (for example, Diabetes/Perinatal Mental Health/Obstetric High-Risk Clinics) are attended virtually. +/− Remote self-monitoring or Telemonitoring of fetal/maternal parameters.
Inpatient/Hospital at Home settings	Admission to hospital for increased monitoring is replaced with telemonitoring monitoring at home, where the monitoring systems communicate directly with the clinicians electronically (e.g., with a software application that is linked to the remote monitoring equipment) and communication with clinicians is done by video or telephone.

### 2.5. Data Synthesis

Data were extracted from the 21 studies and analysed using seven-phase meta-ethnography approach of [10]. Three members of the author team independently read and re-read each study to enable deep immersion in the data and identify key first-order (participant accounts) and second-order (author interpretations) constructs. Studies were then examined comparatively to determine how their key concepts and themes related to one another, including identifying recurring metaphors, patterns, and interpretive constructs across the dataset.

Reflexive discussions were held across the multidisciplinary author team to challenge assumptions, compare interpretations, and ensure that no single study or disciplinary perspective disproportionately shaped the synthesis. Where differences in interpretation arose, these were resolved through return to the original study texts, explicit comparison of alternative interpretations, and assessment of conceptual fit across studies, rather than by consensus alone. The author team’s professional backgrounds, primarily in midwifery, maternity care, and maternal and child health, alongside one obstetrician, informed sensitivity to relational care, safety, and equity, while reflexive dialogue was used to critically examine how these professional lenses influenced analytic decisions.

Through a process of reciprocal translation, concepts from each study were compared and reinterpreted in the context of others, preserving their original meanings while enabling cross-study integration. Translated concepts were synthesised to generate high-order interpretations by reinforcing shared understandings (reciprocal synthesis), highlighting contradictions (refutational synthesis), or constructing a broader interpretive framework (line-of-argument synthesis). This process produced a coherent conceptual narrative that extends beyond individual studies, offering a deeper understanding of how pregnant women experience VMC models of antenatal care.

## 3. Results

Following quality appraisal using the Critical Appraisal Skills Programme (CASP) qualitative checklist, all 21 included studies were judged to demonstrate sufficient methodological rigour to contribute meaningfully to the interpretive synthesis and were retained for analysis.

The synthesis of qualitative studies found that women’s experiences with virtual maternity care are complex, shaped by service design and delivery. Four main themes were synthesised from the authors’ analyses and participant data included in the papers: ‘*Virtual Care Worked Well*’, ‘*Seeking Good Connections*’, ‘*Empowerment and Safety Through Virtual Care Monitoring*’, and ‘*Feeling Disconnected and Unsafe: When Virtual Care Failed to Support*’.

These findings highlight both the benefits and the risks of virtual maternity models, which are explored in detail below.

### 3.1. Virtual Care Worked Well

The first theme, ‘*Virtual Care Worked Well*’, captures how many women experienced virtual maternity care as positive and beneficial for themselves and their families [4,5,18,19,22,23,25,26,29,33]. Across studies, women framed virtual care as enabling not simply because it replaced in-person visits, but because it reduced the practical and emotional labour associated with accessing antenatal services. Convenience and flexibility were the dominant qualities underpinning these positive experiences.

Virtual care was consistently valued for minimising time and effort, allowing women to integrate pregnancy care more easily into daily life [4,18,19,23,26,33]. Avoiding travel, time away from work, and waiting rooms offered a sense of freedom and control over how care was accessed. As one participant described, virtual appointments were simply, “*quick, easy, fast*” [17] (p. 643), while another reflected that “*the days when I don’t have to go to the hospital. I have a little more freedom*” [29] (Catherin, p. 7). Technologies that were intuitive and reliable further supported these experiences, particularly cellular-enabled devices that did not rely on home internet access and could be used, “*I was able to take it everywhere with me*” [20] (p. 1195).

Flexibility emerged as a key enabler of access, particularly for women balancing paid work, family responsibilities, and health needs [4,5,17,19,22,25,29,30,33]. The ability to schedule appointments around other commitments, attend consultations during work hours, or switch between video and telephone modalities reduced missed visits and increased engagement with care. For some women, this flexibility was critical to participation, as one Hispanic/LatinX participant explained, “*There are moms that sometimes don’t have the possibility to go… and they can ask permission at work to make a phone call and they tell them, ‘of course’*” [17] (p. 651).

The cumulative effect of convenience and flexibility translated into tangible savings in time, money, and energy [19,22,23,25,28,29,33]. Women described reduced travel costs, less reliance on childcare, and fewer disruptions to employment, making antenatal care feel more sustainable over the course of pregnancy. As one woman noted, “*Honestly, I probably would opt for virtual care, just because I mean, gas prices are going up*” [19] (p. 3). Beyond individual benefits, these efficiencies extended to partners and families, reducing disruption to routines and supporting overall wellbeing [7,29,32].

Virtual care was also associated with enhanced perceptions of safety, particularly during the COVID-19 pandemic, when women sought to minimise exposure to infection in clinical settings [4,27]. Remaining at home provided both physical and psychological reassurance, with one participant describing telemedicine as offering “*a security… I can stay at home with my own germs*” [7] (Participant 14, p. 4). Psychological safety was also important, with women reporting greater ease discussing sensitive issues in familiar surroundings [22,29]. For some accessing perinatal mental health support, telephone consultations were preferred over video, offering a sense of privacy and emotional comfort: “*I found it easy to speak to her [the psychologist] over the phone… freedom to speak freely without feeling judged*” [26] (Participant 1, p. 7).

### 3.2. Seeking Good Connections

The second theme, ‘*Seeking Good Connections*’, describes how important it was for women to experience a sense of connection when accessing care virtually [4,5,16,18,21,22,23,24,26,27,32,33]. Firstly, this included the woman’s ability to confidently use the technology. In-person training played a vital role in building this confidence, helping women understand how to operate, manage, and troubleshoot devices [4,27,29,32]. Face-to-face instruction allowed women to ask questions and feel assured in their ability to use the equipment correctly: “*When they first gave me the kit to do it at home, we spent quite a while going through how to put the cuff on, so I feel very confident that I’m doing it correctly*” [27] (Participant 15, p. 10). Trust in the technology itself was also crucial. Women valued accurate and reliable devices, which enhanced their sense of safety and engagement with care. “*I find it quite reassuring… I’ve found it quite a positive experience and I think it is quite good*” [25] (Participant S12). In contrast, unreliable equipment undermined confidence: “*If the machine [cellular blood pressure cuff] was accurate, then I would feel more comfortable taking readings, but I stopped using it because it wasn’t accurate*” [21] (Participant, p. 161).

Beyond technological reliability, feeling personally connected to the healthcare team was central to women’s sense of safety and trust in virtual care. Several constructs were important here, beginning with the understanding that pregnancy was viewed by women as a deeply personal, meaningful, and unique life event [4,18,32]. Therefore, virtual care models that supported continuity and fostered relationships were preferred [4,5,27,32]. Continuity of care frameworks enhanced satisfaction and trust, particularly when women had ongoing relationships with known caregivers [5,29,30,33]: “*It felt very safe… They know who I am, they have my journal… The whole bit can be a stress otherwise*” [30] (Woman no 7, p. 4). Frequent, responsive communication further strengthened trust in the care team: “*I really appreciated the daily phone calls… the midwife functioned as a sympathising listener… They were really helpful*” [32] (TM01, p. 6). In virtual care settings, personalised and relational approaches helped women feel seen, heard, and entitled to care, particularly important for those navigating complex or marginalised circumstances [4,24,28]. Similarly, the negotiation of candidacy that occurs between women, their caregivers and the healthcare system throughout the remote care process, enabled women to feel equally involved in navigating their pregnancy care [24].

### 3.3. Empowerment and Safety Through Virtual Care Monitoring

When virtual care was tailored to women’s individual needs, supported by trusted clinicians, and enabled meaningful engagement with health data, it fostered a sense of safety, autonomy, and control [21,23,27,29,31,32]. Central to women’s experiences of virtual antenatal care was the balance between clinical oversight and personal responsibility. Seamless access to trusted care providers was a critical factor underpinning women’s confidence in assuming monitoring responsibilities [7,21,27,29,31]. The reassurance provided by clinician oversight was highlighted by the following woman: “*I’m not a professional… therefore it’s very nice that I submit the information and they [the clinicians] can log on a computer and see it*,” [7] (P1, p. 4). Similarly, access to a 24 h pregnancy advice line enhanced communication and responsiveness: “*Wherever I’ve had a worry I’ve been able to call that number*” [5] (W35, p. 7). When remote self-monitoring (woman collects and reports their own measurements) was framed as a collaborative activity, women reported feeling supported and safe [7,23,31]. Many appreciated the opportunity to share responsibility for monitoring their health with trusted care providers, contributing to a sense of security and confidence, a feeling described by one participant as “*peace of mind*” [23] (Participant A9, p. 884).

Engaging directly with health data through remote monitoring fostered deeper understanding of physical conditions and health needs [21,31]. Many women found objective data more reliable than symptomatic assessments: “*It provided objective information to really judge it… I find it difficult to determine what is the matter, simply by how I feel*” [31] (Participant 8, p. 9). Others described how monitoring helped them recognise subtle changes, such as: “*It helped me to monitor my numbers to make sure that everything was OK and it was helping me to kind of keep a gauge on my blood pressure*” [21] (p. 160). This increased awareness helped women feel more in control and better equipped to manage their pregnancies [7,21,25,27,29]. Importantly, women’s sense of safety and confidence was not derived from self-monitoring alone, but from the knowledge that readings were reviewed by clinicians, supported by clear escalation pathways and access to timely professional advice when concerns arose. As women gained knowledge through monitoring, many described becoming more competent in managing their health, with increased confidence and self-efficacy [7,18,21,23,27,29,31]. Women managing conditions such as hypertension or gestational diabetes reported being able to identify triggers and make informed lifestyle changes, sometimes leading to lasting behaviour change, “*Because I was able to monitor it myself, I could kind of pinpoint what I needed to eat and what I didn’t need to eat for that day. So that takes a lot of stress off too*” [21] (p. 160).

For women experiencing pregnancy complications, virtual care also offered a sense of normalcy and control that helped reduce feelings of stigma and shame. Managing their condition from home contributed to a more positive perception of pregnancy: “*Not going to the hospital so often makes me regain a feeling of having a normal pregnancy*” [29] (Catharin, p. 8). In the face of uncertainty, virtual care helped women feel more in control: “*There’s so much in pregnancy that you’re not in control of… it’s quite nice to be in control of that*” [27] (Participant 15, p. 10/Appendix 4).

### 3.4. Feeling Disconnected and Unsafe: When Virtual Care Failed to Support

When virtual maternity care was poorly implemented, women described feeling isolated, anxious, and unsupported. Inconsistent data accuracy made women feel that they could not trust the care they were receiving, “*It gave three quite wildly different readings so I felt like I couldn’t trust it*” [25] (Participant S49). Slow transmissions inhibited quality of care, ease and convenience of use [23]. Those that struggled with technology found that it decreased their confidence in their virtual care delivery. If the technology failed, women became anxious and experienced a loss of control, as one participant expressed, “*When some of the measurements disappeared, I could feel that, uh, my heart rate went up sky high and I just thought ´really, what the hell is going on?*” [7] (P4, p. 5).

Poor reception and connection dropouts in some locations restricted women’s access to quality interactions during appointments [23,26,33], for example, “*… if it cuts in and out while you’re trying to have a meaningful conversation, it can be frustrating*” [19] (p. 4). This impacted women in some rural and remote areas who regularly experienced interruptions to their internet service [5,22,29]. One participant shared, “*I sometimes have to stand in the middle of the street for a signal which isn’t ideal when talking about private issues*” [33] (p. 425).

Digital poverty and/or financial barriers limited women’s access to technology, preventing their connection with and inclusion in models of virtual care. Groups that were especially vulnerable were those who were digitally excluded through lack of internet access (often occurring for women in rural areas), lack of hardware or mobile data or those who had low levels of digital literacy [24]. The studies clearly highlighted that virtual care did not necessarily make quality care more accessible to all women, generating the potential to increase inequities in care [4,5,22,33].

In studies conducted during the rapid implementation of virtual maternity care during the COVID-19 pandemic, many women reported feeling disconnected from their care providers and unsupported by the system, often feeling “*forgotten*” [33] (No. 2, p. 422) and as if they were navigating their pregnancy care alone. Poorly integrated services and fragmented communication left women feeling as though they had to “DIY” their care, generating anxiety about the potential consequences for their unborn baby if something important was missed. A common issue across these settings was the lack of interoperability between maternity systems, creating siloed communication between providers. Incompatibilities between software platforms and incomplete digitisation of medical records meant that women often had to take on administrative and technological responsibility for tracking their own health information [24]. In many cases, women held more up-to-date information than their clinicians could access, shifting the burden onto them to flag concerns and share relevant data: “*I keep a separate […] paper diary, where I just log all of my appointments, and test results that I’ve got access to and, so I can just keep track of everything in one place as well*” [24] (W05, p. 227). This breakdown in system coordination made it difficult for some women to advocate for themselves or negotiate their care. Authors described this as a challenge to “assert candidacy” [24] (p. 225). The following two participant extracts from the work of [24] support this assumption: “*I didn’t want to sound paranoid just to call them and say I’m really worried I haven’t seen anyone… I just tried to keep calm but it’s hard*” (W30, p. 225) and “*Because I was always wondering if I was missing something, should I have made an appointment, should I have done this, should I have done that*” (W20, p. 225).

The burden of self-monitoring without adequate support was another major concern. Women who received equipment such as blood pressure monitors or Dopplers without accompanied training often felt ill-equipped to use them, fearing they might misinterpret results or overlook signs of complications [18,25,33], “*So, they gave me a blood pressure monitor and a Doppler. So, I guess, I’m going to be in charge of doing those things, which is making me nervous*” [18] (G1, Patient 2, p. 5).

Studies undertaken during the COVID-19 pandemic provided insight into the inhibitors or barriers to facilitating team connections. Virtual care that lacked relationality or felt rushed was problematic [4,5,16,24,33]. When virtual care appointments were hurried, women reported feeling ignored or “*brushed over*” [5] (W29, p. 6). Consequently, many women perceived they were not receiving the level of care they deserved during pregnancy. Authors in [33] described this as “*feeling let down by the maternity care system*” (p. 425). Some women described their virtual care experiences as feeling too formal, “*like a business interaction*” [5] (W94, p. 7) or, as noted by [26], “*less personal*” (p. 6). Women also reported feeling like their telehealth (synchronous or asynchronous consultations delivered via phone or video) appointments were perfunctory; “*I sometimes feel like it’s just kind of checking those boxes off*” [16] (C2, p. 46). Some interactions were described as transactional, and protocol driven, rather than therapeutic [5,16,24]. Focusing solely on the clinical aspects of care at the expense of the relational qualities left women feeling disconnected [4].

Finally, when women using telemonitoring (defined here as the automatic transmission of physiological data to clinicians) at home experienced delays in receiving feedback from their virtual care team regarding the outcome of their readings their anxiety was heightened [7,29]; “*I am simply afraid of putting my phone away or on silent*” [29] (Catherin, p. 9). Another woman described the experience of long waiting times for reassurance as being in “*no man’s land*” [29] (Tanja, p. 9).

## 4. Discussion

This metasynthesis draws on 21 qualitatively appraised studies synthesising women’s experiences of virtual maternity care across antenatal settings in high-income countries. While reporting depth and methodological transparency varied, all studies demonstrated sufficient rigour to inform interpretive synthesis, with considerations of quality guiding reflexive interpretation rather than exclusion. Accordingly, the deliberate distinction between telehealth, remote self-monitoring, and telemonitoring in this metasynthesis reflects women’s differing experiences of interaction, responsibility, and relational support across virtual care modalities, rather than interchangeable technological functions.

The findings of this metasynthesis demonstrated that virtual antenatal care can be either empowering or problematic. While many included studies were conducted during the COVID-19 pandemic, the synthesis distinguishes pandemic-specific disruptions from enduring relational, technological, and equity considerations relevant to post-pandemic virtual maternity care. Virtual models can greatly enhance access, convenience, and the woman’s engagement, often improving clinical outcomes and efficiency, so long as they are implemented in a way that preserves relationship-based care and equitable inclusion. The implication is that health systems should embrace virtual care as a complementary part of maternity services while investing in strategies to maintain personal connection, support maternal self-efficacy, and avoid digital exclusion.

Women valued the convenience of virtual antenatal care, the reduced travel time, avoidance of waiting rooms, and feeling safer at home [17,22,33]. These findings align with the work of others who have shown that virtual antenatal care can improve access to care and reduce burdens on women and health systems without compromising quality [52]. For example, in the Netherlands, Bekker, et al. found that high-risk pregnant women using home telemonitoring had similar or better clinical outcomes to those admitted for in-hospital monitoring, with greater satisfaction and lower costs [2].

Likewise, a 2025 scoping review of 126 studies noted high acceptance, satisfaction and positive outcomes with digital antenatal interventions, including improved management of conditions like diabetes and hypertension, fewer in-person visits, and substantial cost savings for women and providers [9]. These findings underscore that virtual care, when properly implemented, works well not just as a pandemic stopgap but as a sustainable model to enhance efficiency and access in maternity care.

A key finding from this metasynthesis was that human connection, via both technological connectivity and caregiver relationships, is at the heart of quality maternity care. Feeling known, understood, and supported by care providers was closely tied to women’s sense of safety and trust [31,32]. Virtual models that fostered continuity, particularly when built on existing in-person relationships, were more likely to be experienced as reassuring and effective [4,5,32]. Hybrid models, which blend virtual and face-to-face interactions, were consistently preferred, as they allowed women to establish rapport, ask questions comfortably, and feel confident in their care. These findings strongly resonate with the results of an extensive scoping review of digital technologies in antenatal care undertaken by [9]. These authors concluded that hybrid models, where digital technologies were complementary to standard care, allowed services to adapt to individual needs while maintaining continuity and emotional safety, making them a critical component of equitable and effective virtual care design. This supports the wider evidence that relationship-based care is pivotal in maternity services. Research on continuity of midwifery care, for example, shows that having the same midwife or small team of midwives throughout pregnancy leads to better outcomes and experiences, including higher rates of vaginal birth, lower intervention rates, and greater satisfaction [53]. Importantly, the reliance on relational continuity and hybrid flexibility observed here may represent a comparatively advantaged experience; in contexts of digital exclusion, linguistic barriers, or psychosocial vulnerability, the absence of in-person options and consistent caregiver relationships may further compound inequities in virtual maternity care access and safety. In virtual settings, maintaining a personal rapport requires deliberate effort. Professional bodies, such as [54] emphasise that virtual care should complement, not replace, in-person woman centred care.

Women described feeling empowered when they could actively participate in monitoring their own and their baby’s health and wellbeing (for example, checking their blood pressure or baby’s heart rate at home) with oversight from clinicians [21,27,29,31]. This speaks to a broader trend in healthcare: engaging women as partners can improve outcomes [55,56] and shows, like others, that remote monitoring programs in pregnancy not only make women feel more in control but can objectively improve clinical management [57]. For instance, telehealth interventions have helped women with gestational diabetes and hypertension achieve better disease control with fewer clinic visits, by enabling daily home measurements and timely adjustments to therapy [2]. Likewise, Mohamed et al. [9] found that digital health interventions in pregnancy were consistently associated with improved maternal understanding of health and the promotion of healthier behaviours. In their scoping review, engagement with telehealth platforms and mobile applications was linked to improved recognition of warning signs and higher levels of maternal health literacy, findings that align closely with those of the present metasynthesis. Importantly, women often translated this increased knowledge into positive actions, such as improved diet, appropriate weight gain, and adherence to care recommendations. Similarly, in the UK, women who self-monitored their blood pressure (BP) at home consistently reported that tracking their own BP readings gave them a much deeper understanding of their condition [58]. Women learned how their blood pressure fluctuated with daily activities and stressors, an insight they would not have gained from infrequent clinic checks. This ‘embodied knowledge’ made women more confident in managing their pregnancy health. This approach is particularly useful for women who experience hypertensive disorders of pregnancy, as these conditions are associated with substantially increased long-term risks of cardiovascular, renal, and metabolic disease [59,60]. However, structured follow-up beyond the immediate postnatal period is frequently limited due to fragmented models of care, inconsistent awareness among women and healthcare providers regarding long-term cardiometabolic risk, and uncertainty about responsibility for ongoing monitoring and prevention [61,62,63]. As a result, opportunities for timely surveillance and preventive health interventions are often missed, despite well-established evidence that pregnancy complications mark increased future risk of cardiovascular and metabolic disease and warrant risk-informed follow-up.

Consistent with our metasynthesis, studies [61,62,63] show that women using telehealth or mobile platforms during antenatal care demonstrated improved recognition of warning signs and higher health literacy. While evidence of sustained long-term disease prevention remains limited, these findings highlight the potential for pregnancy-based learning to act as a foundation for later health-protective behaviours, supporting growing calls to conceptualise pregnancy as a critical window for engaging women in life-course cardiovascular and metabolic risk awareness.

Overall, research suggests that integrating self-monitoring and education with technology in prenatal care can inform, empower and motivate women to take charge of their own health, while maintaining similar clinical outcomes to traditional care [9]. However, the literature also cautions that such empowerment works best when there is responsive clinical support behind it [64]. Women derive ‘peace of mind’ knowing that in an event of a concerning reading, a midwife or doctor is immediately available to provide guidance; essentially sharing responsibility for safety. Across the studies included in this metasynthesis, this reliance on timely clinical backup was evident in both emergency pandemic implementation and more established models of virtual care, although disruptions to continuity and responsiveness were more pronounced during periods of rapid, crisis-driven rollout. The findings of the metasynthesis demonstrated that it is this combination of maternal autonomy and known provider backup that makes remote monitoring a confidence-building rather than anxiety-provoking experience.

Not all experiences with virtual maternity care were positive; some women, particularly those with prior pregnancy loss, mental health issues, or intimate partner violence, felt isolated, anxious, or poorly cared for in fully virtual models [26,28]. These concerns are echoed in broader discourse: experts caution that certain signals and needs can be missed when care is remote, and that virtual care can inadvertently exacerbate disparities if not implemented carefully [9]. For instance, in telehealth encounters it may be harder to pick up non-verbal cues of distress or to conduct private screening for domestic violence. A controlling partner might be just off-screen, making it challenging for providers to ensure the woman’s safety [28,65]. Likewise, women with limited English proficiency, low digital literacy, or no internet access may find virtual care alienating or inaccessible [4,33,66]. These digital divide issues are well documented: lack of reliable internet or comfort with technology disproportionately affects older, lower-income, and minority women, potentially widening healthcare gaps if virtual care becomes the default [9,67]. Simply put, a ‘one-size-fits-all’ fully virtual model could leave vulnerable woman feeling disconnected and underserved, exactly the opposite of providing quality woman centred maternity care.

### 4.1. Implications

#### 4.1.1. Theoretical Implications

The findings of this metasynthesis contribute to socio-technical theories of digital health by demonstrating that virtual maternity care is experienced by women not as a neutral technological intervention, but as a relational practice embedded within specific models of care. Consistent with Greenhalgh and colleagues’ conceptualisation of digital health as a socially and technically mediated form of care, women’s perceptions of safety, reassurance, and quality were shaped less by the technology itself and more by how virtual tools were integrated into relational midwifery models [34,68]. From this perspective, safety is not an inherent property of digital platforms, but an emergent feature of socio-technical systems in which relationships, organisational arrangements, and technologies interact.

The findings also extend theories of continuity of care, particularly relational continuity, which has long been identified as central to trust, sense-making, and perceived safety over time [68]. Drawing on this framework and its later extension in digital contexts [69] the metasynthesis illustrates that virtual models may preserve informational and management continuity while simultaneously placing relational continuity at risk when care becomes fragmented or episodic. Women’s accounts suggest that continuity is experienced not merely as consistency of personnel, but as an ongoing relational process through which safety and reassurance are negotiated. In this way, the metasynthesis advances continuity theory by illustrating how continuity is felt and enacted by women within virtual maternity care, rather than simply organisationally achieved.

#### 4.1.2. Implications for Virtual Maternity Care Practice

To ensure continuity in virtual care, it is important to assign every woman a dedicated midwife or small team who remains consistent throughout their care. Early in-person visits can build trust before transitioning to virtual formats. Providers need training in ‘webside manner’, and women benefit from onboarding sessions to build confidence with devices and apps. Reliable, user-friendly platforms and access to equipment (for example, loaned devices, data plans) are essential for maintaining connection and inclusion.

Women should be equipped with home monitoring tools and clear guidance in relation to receiving their results. Clinicians can help women understand health patterns and make lifestyle changes. Safety protocols, like automatic alerts and 24/7 contact lines, ensure women are never left alone with concerning data. Remote monitoring must be supported by staffing, liability coverage, and integration with medical records.

Finally, hybrid models (virtual and in-person) are preferable for most pregnancies. Care plans should be tailored; some women need more in-person support due to clinical or psychosocial risks. Technology access must be addressed through device loans, data support, and flexible communication options. Ongoing evaluation and feedback mechanisms are vital to ensure that virtual maternity care remains safe, equitable, and responsive to the evolving needs of women. This approach supports a high-quality service that adapts to feedback and continues to improve in line with women’s experiences and expectations.

### 4.2. Strengths and Limitations

This metasynthesis offers a rich, interpretive synthesis of women’s experiences with virtual maternity care, drawing on 21 qualitative studies conducted across high-income health system settings. The use of meta-ethnography enabled deep conceptual integration while preserving the nuance and contextual specificity of individual accounts. By centring women’s voices, the synthesis foregrounds relational, emotional, and practical dimensions of virtual care that are often under-examined in service evaluations focused primarily on efficiency or clinical outcomes.

However, the findings are limited by the exclusion of studies from low-resource settings and by the underrepresentation of women experiencing digital exclusion, cultural and linguistic marginalisation, or structural disadvantage within the primary studies themselves. The exclusion of low- and middle-income countries was a deliberate methodological decision to support conceptual coherence within the meta-ethnographic synthesis, given that virtual maternity care is strongly shaped by health-system infrastructure, digital connectivity, workforce configuration, and models of service delivery that differ substantially across economic and policy contexts. These limitations are not merely methodological constraints but shape how the findings should be interpreted, particularly in relation to equity and access within emerging virtual maternity care systems.

Variability in terminology, service configuration, and the rapid implementation of virtual care models, particularly during the COVID-19 pandemic, may also have influenced interpretation across studies. Importantly, these limitations do not negate the findings but instead illuminate persistent gaps in the evidence base, underscoring the need for future research that explicitly centres equity, inclusion, and the relational dimensions of virtual maternity care in diverse contexts.

## 5. Conclusions

This metasynthesis demonstrates that Virtual Maternity Care can support women’s engagement, autonomy, and perceived safety when models are thoughtfully designed and implemented. Women valued the convenience and flexibility of virtual care, particularly when it was underpinned by relational continuity, reliable technology, and access to trusted clinicians. However, poorly integrated or exclusively virtual models risk undermining safety and amplifying inequities, especially for women experiencing psychosocial vulnerability or digital exclusion. These findings highlight the importance of hybrid, relationship-centred models of virtual maternity care that prioritise equity, continuity, and responsiveness. Healthcare leaders and policymakers should ensure that digital innovation in maternity services strengthens, rather than fragments, care, by embedding relational practice, inclusive design, and flexible delivery within evolving virtual care systems.

## Data Availability

This article is a metasynthesis of published literature, no new data were created in this study, hence, data sharing is not applicable.

## Supplementary Materials

The following supporting information can be downloaded at: https://www.mdpi.com/article/10.3390/ijerph23050607/s1, File S1: PRISMA Checklist; File S2: Search Strategy.
