# Supplementary material for: Virtual Maternity Care During Pregnancy: A Metasynthesis of the Qualitative Literature on Women’s Experiences
Source: Int J Environ Res Public Health. 2026 May 4;23(5):607. doi: 10.3390/ijerph23050607 (PMC13207183; doi:10.3390/ijerph23050607)
Supplement: Supplementary file 1 [file ijerph-23-00607-s001.zip › ijerph-4239747-supplementary.pdf]

PRISMA 2020 Checklist

Manuscript: Virtual maternity care during pregnancy: a metasynthesis of the qualitative literature on women's experiences

Journal: International Journal of Environmental Research and Public Health (IJERPH)

Manuscript ID: ijerph-4239747v1

This checklist documents where each PRISMA 2020 item is addressed in the manuscript or indicates where items are not applicable for a qualitative metasynthesis using meta-ethnography.

Section & Topic	Item#	Checklist Item	Location reported
Title	1	Identify the report as a systematic review	Title page
Abstract	2	See the PRISMA 2020 for Abstracts checklist	Abstract pp. 1-2
**Introduction**			
Rationale	3	Describe the rationale for the review in the context of existing knowledge	Introduction, pp. 2–3
Objectives	4	Provide an explicit statement of the objective(s) or question(s) the review addresses	End of Introduction, p.3
**Methods**			
Eligibility criteria	5	Specify the inclusion and exclusion criteria for the review	Methods – Inclusion and Exclusion Criteria, p.4
Information sources	6	Specify all information sources and dates searched	Methods – Search Strategy, p.3
Search strategy	7	Present full search strategies for all databases	Supplementary File S1 (Search strategies)
Selection process	8	Specify methods used to decide study eligibility, number of reviewers, and independence	Methods – Search Strategy and Screening Process, pp.3-5

Data collection	9	Specify methods used to collect data from reports	Methods – Data Characteristics, p.5
Data analysis	10a	List and define all outcomes for which data were sought	Methods – Data Characteristics and Synthesis (qualitative focus) pp.5, 12
Data analysis	10b	List and define other variables for which data were sought	Methods – Data Characteristics, Data Synthesis, pp. 5, 12; Tables 1–3
Study risk of bias assessment	11	Specify methods used to assess risk of bias / quality	Results – Quality Appraisal (CASP), p.12
Effect measures	12	Specify effect measures used	Not applicable – qualitative metasynthesis
Synthesis methods	13a	Describe processes used to decide which studies were eligible for each synthesis	Methods – Data Synthesis, p.12
	13b	Describe methods used to prepare data for synthesis	Methods – Data Synthesis, p.12
	13c	Describe methods used to tabulate or visually display results	Tables 1–3; Figure 1
	13d	Describe methods used to synthesise results and rationale	Methods – Data Synthesis (meta-ethnography), p.12
	13e	Describe methods to explore heterogeneity	Not applicable – qualitative metasynthesis
	13f	Describe sensitivity analyses	Not applicable – qualitative metasynthesis
Reporting bias assessment	14	Describe methods used to assess reporting bias	Not applicable – qualitative metasynthesis
Certainty assessment	15	Describe methods used to assess certainty/confidence in the body of evidence	Not applicable – qualitative metasynthesis

**Results**			
Study selection	16a	Describe results of the search and selection process	Results – Screening description; Figure 1 PRISMA diagram
	16b	Cite excluded studies and reasons for exclusion	Results – Screening description; Figure 1 PRISMA diagram
Study characteristics	17	Cite each included study and present characteristics	Table 1, pp. 6-9
Risk of bias in studies	18	Present assessments of risk of bias for each study	Results – CASP Quality Appraisal summary, p.12
Results of individual studies	19	Present results of individual studies	Results – Narrative synthesis, pp.12-16; Table 1
Results of syntheses	20a	Summarise characteristics of studies contributing to each synthesis	Results – Theme introductions pp.12-16; Table 1
	20b	Present results of syntheses	Results – Thematic findings pp.12-16; Table 1
	20c	Present results of heterogeneity investigations	Not applicable – qualitative metasynthesis
	20d	Present results of sensitivity analyses	Not applicable – qualitative metasynthesis
Reportig biases	21	Present assessments of reporting bias	Not applicable – qualitative metasynthesis
Certainty of evidence	22	Present assessments of certainty/confidence in the evidence	Not applicable – qualitative metasynthesis
**Discussion**			
	23a	Provide a general interpretation of the results	Discussion, pp.16-20
	23b	Discuss limitations of the evidence	Strengths and Limitations, pp.19-20
	23c	Discuss limitations of the review processes	Strengths and Limitations, pp.19-20

	23d	Discuss implications for practice, policy, and research	Implications section, p.19
**Other information**			
Registration and protocol	24a	Provide registration information or state not registered	Methods – Reporting Standards statement
	24b	Indicate where protocol can be accessed or state none prepared	Methods – Reporting Standards statement
	24c	Describe amendments to protocol	Not applicable – no protocol
Support	25	Describe sources of support and role of funders	Funding section, p.20
Competing interests	26	Declare competing interests	Conflicts of Interest Statement, p.21
Availability of data	27	Report availability of data and materials	Data Availability Statement, p.20

Note: Items marked Not applicable reflect the qualitative meta-ethnographic design and are consistent with PRISMA 2020 guidance for non-meta-analytic systematic reviews.
