# Supplementary material for: Virtual Maternity Care During Pregnancy: A Metasynthesis of the Qualitative Literature on Women’s Experiences
Source: Int J Environ Res Public Health. 2026 May 4;23(5):607. doi: 10.3390/ijerph23050607 (PMC13207183; doi:10.3390/ijerph23050607)
Supplement: Supplementary file 1 [file ijerph-23-00607-s001.zip › Supplementary_File_S2_ Search Strategy.pdf]

Title: Virtual maternity care during pregnancy: a metasynthesis of the qualitative literature on women's experiences

Inclusion and Exclusion Criteria:

Inclusion	Exclusion
English Language	Not available in English
Published since 1st January 2010	Published before 2010
Antenatal period	Intrapartum only
Antenatal period: Pre-labour or induction of labour (discharged home)	Postnatal only
Primary Research	Not primary research
Full text available	Full text not available
Qualitative (and qualitative data from mixed methods)	Quantitative only
Women's experiences	Healthcare providers experiences only

Dates

Included range: 1st January 2010 – 3rd June 2025
Search: 3rd June 2025

Results

MEDLINE: 783
EMBASE: 1182
CINAHL: 2049
SCOPUS: 1182

SUB TOTAL = 5208
After duplicates (1874) removed = TOTAL 3334

Medline/Embase Search Strategy:

Virtual	Maternity care	Experience	Women/Pregnant People
exp telehealth/<br>Telemedicine/<br>Distance counselling/<br>Mental health teletherapy/<br>Telepathology/<br><br>Virtual.mp<br>Virtual hospital.mp<br>Telehealth.mp<br>Telemedicine.mp<br>Remote.mp<br>Telemonitoring.mp<br>Ehealth.mp<br>E-Health.mp<br>Mhealth.mp<br>Videoconferenc*.mp	Exp Maternal Health Services/<br>Midwifery/<br><br>Antenatal care.mp<br><br>Care adj3 (maternity or prenatal or perinatal or antenatal or pregnancy or maternal or antepartum or fetal or foetal or pregnant)<br><br>healthcare adj3 (maternity or prenatal or perinatal or antenatal or pregnancy or maternal or antepartum or fetal or foetal or pregnant)	Exp personal satisfaction/<br><br>Experience*.mp<br>Satisfaction.mp<br>Attitude.mp<br>Perspective*.mp<br>View*.mp<br>Emotional wellbeing.mp<br>Perception*.mp	Exp Parents/<br><br>Wom?n*.mp<br>Pregnant people*.mp<br>Pregnant person*.mp<br>Birthing people*.mp<br>Birthing person*.mp<br>Famil*.mp<br>Mother*.mp<br>User*.mp

	Service* adj3 (maternity or prenatal or perinatal or antenatal or pregnancy or maternal or antepartum or fetal or foetal or pregnant)<br><br>Provi* adj3 (maternity or prenatal or perinatal or antenatal or pregnancy or maternal or antepartum or fetal or foetal or pregnant)<br><br>Monitor* adj3 (maternity or prenatal or perinatal or antenatal or pregnancy or maternal or antepartum or fetal or foetal or pregnant)		

CINAHL Search Strategy:

**Virtual**	**Maternity care**	**Experience**	**Women/Pregnant People**
(MH"Home Health Care+")<br>(MH"Digital Health+")<br>(MH"Telemedicine+)<br>OR (MH "Telehealth+")<br>(MH"Videorecording")<br><br>"Virtual"<br>"Virtual hospital"<br>"Telemedicine<br>"Remote"<br>"e-health"<br>"ehealth"<br>"mhealth"<br>"Videoconfer*"	(MH "Maternal-Child Care+")<br>MH "Maternal Health Services+")<br>MH Midwifery+<br>MH Pregnancy+<br><br>"Antenatal care.mp"<br><br>Care n3 (maternity or prenatal or perinatal or antenatal or pregnancy or maternal or antepartum or fetal or foetal or pregnant)<br><br>healthcare n3 (maternity or prenatal or perinatal or antenatal or pregnancy or maternal or antepartum or fetal or foetal or pregnant)<br><br>Service* n3 (maternity or prenatal or perinatal or antenatal or pregnancy or maternal or antepartum or fetal or foetal or pregnant)	(MH"Attitude+")<br>(MH"Personal Satisfaction")<br>(MH"Emotions+"<br><br>"experience*"<br>"satisfaction"<br>"attitude"<br>"perspective*"<br>"view*"<br>"emotional wellbeing"<br>"perception"	(MH"Parents+")<br>(MH"Women+")<br><br>"women*"<br>"woman*"<br>"pregnant people*"<br>"pregnant person"<br>"birthing people"<br>"birthing person*"<br>"famil*"<br>"mother*"<br>"user*"

	Provi* n3 (maternity or prenatal or perinatal or antenatal or pregnancy or maternal or antepartum or fetal or foetal or pregnant)<br><br>Monitor* n3 (maternity or prenatal or perinatal or antenatal or pregnancy or maternal or antepartum or fetal or foetal or pregnant)		

Scopus Search Strategy:

"virtual" OR "virtual hospital" OR "telehealth" OR "telemedicine" OR "remote" OR "telemonitoring" OR "ehealth" OR "e-health" OR "mhealth" OR "videoconference"

AND

"maternal health services" OR "antenatal care" OR "midwifery" OR (care W/3 (maternity OR prenatal OR perinatal OR antenatal OR pregnancy OR maternal OR antepartum OR fetal OR foetal OR pregnant)) OR (healthcare W/3 (maternity OR prenatal OR perinatal OR antenatal OR pregnancy OR maternal OR antepartum OR fetal OR foetal OR pregnant)) OR (service W/3 (maternity OR prenatal OR perinatal OR antenatal OR pregnancy OR maternal OR antepartum OR fetal OR foetal OR pregnant)) OR (provi* W/3 (maternity OR prenatal OR perinatal OR antenatal OR pregnancy OR maternal OR antepartum OR fetal OR foetal OR pregnant)) OR (monitor* W/3 (maternity OR prenatal OR perinatal OR antenatal OR pregnancy OR maternal OR antepartum OR fetal OR foetal OR pregnant))

AND

"experience" OR "satisfaction" OR "attitude" OR "perspective" OR "view" OR "emotional wellbeing" OR "perception"

AND

"woman" OR "women" OR "pregnant people" OR "pregnant person" OR "birthing people" OR "birthing person" OR "famil*" OR "mother" OR "user"
